# Oral diseases and socio-demographic factors in adolescents living in Maasai population areas of Tanzania: a cross-sectional study

**DOI:** 10.1186/s12903-018-0664-6

**Published:** 2018-12-04

**Authors:** Lutango D. Simangwa, Anne N. Åstrøm, Anders Johansson, Irene K. Minja, Ann-Katrin Johansson

**Affiliations:** 1 Department of Clinical Dentistry Cariology, Faculty of Medicine, University of Bergen, Bergen, Norway; 2 Department of Clinical Dentistry Community Dentistry, Faculty of Medicine, University of Bergen, Bergen, Norway; 3 Department of Clinical Dentistry Prosthodontics, Faculty of Medicine, University of Bergen, Bergen, Norway; 4 Department of Restorative Dentistry, School of Dentistry, Muhimbili University of Health and Allied Sciences, Dar Es Salaam, Tanzania

**Keywords:** Adolescents, Dental caries, Dental erosion, Dental fluorosis, Maasai population areas, Oral hygiene, Temporomandibular disorders, Tooth wear

## Abstract

**Background:**

Oral diseases may cause serious health problems, especially in socially disadvantaged populations and in low-income countries. In populations living in the rural areas of Tanzania there is paucity of reports on oral health. The study aim was to estimate the prevalence, severity and socio-demographic distribution of oral diseases/conditions in adolescents living in Maasai population areas of Tanzania and to compare oral diseases/conditions between Maasai and non-Maasai ethnic groups.

**Methods:**

A total of 23 schools were randomly selected from 66 rural public primary schools in Monduli and Longido districts, Tanzania. All pupils in the selected classes, 6th grade, were invited to participate in the study. A total of 989 were invited and 906 (91.6%) accepted the invitation and completed an interview and a clinical oral examination.

**Results:**

Out of 906 study participants (age range 12–17 years), 721(79.6%) were from Maasai and 185 (20.4%) from non-Maasai ethnic groups. Prevalence of poor oral hygiene, gingival bleeding, dental caries experience (DMFT> 0), dental fluorosis TF grade 5–9, dental erosion (into dentin), tooth wear (into dentin) and TMD was 65.6, 40.9, 8.8, 48.6, 1.9, 16.5 and 11.8%, respectively. Multiple variable logistic regression analysis revealed that, girls (OR = 2.0) and participants from Longido (OR = 2.6) were more likely to present with good oral hygiene (*p* < 0.05). Adolescents from Monduli (OR = 1.7), males (OR = 2.1), being born within Arusha region (OR = 1.9) and Maasai (OR = 1.7) were more likely to present with gingival bleeding (*p* < 0.05). DMFT> 0 increased by age (OR = 2.0) and was associated with non-Maasai ethnic group (OR = 2.2), (*p* < 0.05). Adolescents from Monduli district (OR = 10.0) and those born in Arusha region (OR = 3.2) were more likely to present with dental fluorosis (*p* < 0.05). Dental erosion was more common among non-Maasais (OR = 2.0) as well as having mother with high education (OR = 2.3), (*p* < 0.05).

**Conclusions:**

Oral diseases like dental caries and dental erosion were less common, but gingival bleeding, dental fluorosis, tooth wear and TMD were common findings in adolescents attending primary schools in the Maasai population areas of Tanzania. Notable differences between Maasai and non-Maasai ethnic groups and certain correlations to sociodemographic factors were detected. Our findings can be utilized by policy makers in the planning of oral health programs in public primary schools of Maasai population areas of Tanzania.

## Introduction

Oral diseases are among the most common diseases and yet it is not unusual that these type of problems receive little attention especially so in countries with weak health care systems [1]. Most countries in sub-Saharan Africa, for example Tanzania, focus on high mortality diseases like HIV/AIDS, cancer, tuberculosis, diabetes and malaria and pay less attention to oral health issues [2]. In many regions it is therefore common that oral diseases, such as dental caries, are left untreated or at the best treated with tooth extraction as the only choice for emergency treatment [3]. The remote population in Tanzania is more or less excluded from oral health services. To improve the access to general health care for pastoralists in the northern part of Tanzania, to a large extent including the Maasai tribe, village dispensaries/health centers have been placed in specific areas [4]. However, due to the pastoral lifestyle of these groups of people, which implies living and moving together with their animals, this special service has been of limited use [4].

A relation between oral diseases/conditions and sociodemographic factors such as sex, age, education, ethnicity and wealth/family income have been reported worldwide [5–10]. Studies have shown that children from lower-income households are more likely to experience dental caries than their economically more advantaged counterparts [11–13]. It is also clear that children of parents with high education experience less dental caries than children of parents with low education [12, 14]. Considering sex and age, findings from sub-Saharan Africa have shown a consistent pattern in which dental caries experience is not only significantly greater in females, but also increases with age [15–18]. Studies from sub-Saharan Africa have also shown that the prevalence of dental caries often is low and the mean DMFT of 14 year olds living in rural areas of Tanzania (2010), 12 year olds in Kenya (2012), and 10–14 year olds in Uganda (2003) has been found to be 0.3, 0.4 and 0.7, respectively [19–21]. Besides this, one older study as far back as 1931 and another more recent study from 1997 are addressing mainly the medical health of the pastoral societies in Sub-Saharan Africa but also including a few oral health parameters reported that the occurrence of dental caries among the Maasais at that time was lower than in the other groups [22, 23].

Poor oral hygiene have commonly been reported among both children and adolescents and may lead to a number of oral health problems such as dental caries, gingivitis, periodontitis and tooth loss [24, 25]. For example in Zambia, the prevalence of poor oral hygiene in 10–14 year old adolescents (2011) was 10% and in Nigeria the corresponding figures among 11–14 years olds was found to be 32% (2011) [26, 27]. Among adolescents in Tanzania, the prevalence of poor oral hygiene was found to be as high as 65–99% (1988, 1996) and the occurrence of gingivitis as high as 80–90% [28, 29].

Reports from the Eastern part of sub-Saharan Africa show that dental fluorosis is very common and in certain regions its prevalence has been found to be nearly 100% [30, 31]. The prevalence of more severe dental fluorosis according to Thylstrup Fejerskov index (TF-index) grade ≥ 5 in Kenyan adolescents 13–15 year olds was found to be 48% in 2009 [32]. In the Northern part of Tanzania, the concomitant corresponding prevalence in 10–14 year olds (2000) varied between 10 and 34% [33].

The presence of more severe dental erosion in adolescents in many parts of the world has been reported with varying prevalence (3–26%) [34–38]. To our knowledge no studies on dental erosion have been performed in sub-Saharan African adolescents. The literature on tooth wear in general in the Sub-Saharan populations is scarce and focused mainly on adults. A study in the Sudanese population aged 16–75 years and older (2012) found that 26% of the population had tooth wear [39] and a corresponding figure from Nigeria (2010) among of 20–64 year olds was 53% [40].

Temporomandibular disorders (TMD) are a significant public health problem worldwide affecting 3 to 11% depending on diagnosis [41]. Studies on TMD in sub-Saharan Africa are rare, however studies from Nigeria and Tanzania found that 26% of the Nigerian young adults and 67% of the Tanzanian adults had some evidence of signs and symptoms of TMD [42, 43]. Reports from other parts of the world using the research diagnostic criteria (RDC/TMD) are reporting diverging figures. For example among 10–18 year olds, studies in Sweden (1999 and 2009) found that 5–9% of the participants had TMD [44, 45] and in a Saudi Arabian study (2016) it was found that one third of the participants had at least one TMD diagnosis [46]. Among adolescents and young adults in a Mexican study conducted in 2006 it was reported that the prevalence of some grade of TMD was as high as 46% [47].

To the best of our knowledge, there is no information on the oral diseases of contemporary adolescents living in Maasai population areas in Arusha region, northern Tanzania. Thus, the aim of this study was to estimate the prevalence, severity and socio-demographic distribution of oral diseases/conditions in adolescents living in Maasai population areas of Tanzania. It also aimed to explore whether the socio-demographic differences in oral diseases/problems varied according to Maasai and non-Maasai ethnicity.

## Methods

### Sample size

The sample size was estimated based on the assumption that the prevalence of dental erosion among adolescents was 50%. The estimated minimum sample size for this study, 845 adolescents, was obtained by assuming a margin error of 5% and, confidence intervals of 95%. Furthermore, the sample size was multiplied by 2 to account for the design effect (D), and increased by 10% to account for contingencies such as non-response or recording errors.

### Sampling technique

A cross-sectional study was carried out in Maasai population areas of Monduli and Longido districts, in the Arusha region, in the northern part of Tanzania from June to November 2016. The study aimed to focus on 12–14-year-old adolescents, attending rural public primary schools. A list of all primary schools comprising public (urban and rural) and private schools (total of 100 schools) was obtained from both districts. After having excluded urban and private schools, 23 (13 from Monduli and 10 from Longido) out of a total of 66 (38 from Monduli and 28 from Longido) eligible rural public primary schools were randomly selected using a one-stage cluster sample design with school as the primary sampling unit and random number generator software. In each randomly selected school, a class expected to contain adolescents aged 12–14 years was purposively identified (6th grade). All children available in the identified class were invited to participate in the study. Thus, the inclusion criteria were adolescents expected to be in age 12 to 14 year old attending rural public primary schools of Monduli and Longido districts. The exclusion criteria were adolescents attending urban and private primary schools, those absent during the interview/oral examination day and those with learning difficulties.

### Interviews

The questionnaire was constructed in English, translated into Swahili and back-translated to English independently by qualified translators from the University of Dar Es Salaam, Tanzania. Closed- and open-ended questions were used to gather information.

Pre-testing of the questionnaire took place in fifty primary school children (12–14 year olds) before the actual fieldwork regarding wording, meaning, and content of each item, and appropriateness of format and modified as needed. Two especially trained medical nurses performed face-to-face interviews in Swahili/Maa (Maasai language) with each adolescent. Face-to-face interview was done in a school setting, either under the tree or inside the classroom depending on availability and each child was interviewed privately while others were inside their classes. In this study, the pilot participants were not part of the main study.

Socio-demographic factors were assessed in terms of age, ethnicity, sex, place of residence (place of residence was defined as the district where an adolescent was living in e.g., Monduli or Longido districts), father’s and mother’s education, house ownership, number of children, household socio-economic status (perceived affluence of my household) and household wealth index [48]. For the purpose of assessing their wealth index, their livestock were not included because we used Principal Component Analysis (PCA) in calculating the wealth index and PCA works best when the household asset variables are correlated, but also works best when the distribution of variables varies across the households. It is those assets that are more unequally distributed between households that are given more weight in PCA [49]. Variables with low standard deviations carry a low weight from PCA. For example an asset which is owned by almost all households would exhibit no variation between households and would be zero weighted and thus of no use in differentiating the wealth of a particular family. In our study livestock were owned by the majority (93%) of their families, and so livestock was of little or no use in differentiating their wealth. Ethnicity was assessed by asking “what is your ethnic group?” The response categories were (1) = Maasai, (2) = Meru, (3) = Arusha and (4) = others. For analysis, the items were dichotomized to 1 = Maasai (including option (1)) and 2 = non Maasai (including option (2), (3) and (4) during analysis.

Parents’ education was assessed by asking *what is the highest level of school your mother/father has attended?* Responses were (0) for none, (1) for she/he started but did not complete primary school, (2) for completed primary school (3) for she/he started but did not complete secondary school, (4) for she/he completed secondary school, (5) for she/he started but did not complete college/university, (6) for completed college/university, (7) for I don’t know. In the statistical analyses, the items were dichotomized as (0) for low education (from options (0), (1), (2), (3) and (7) and (1) for high education (from options (4), (5) and (6).

Durable household assets indicative of family wealth (i.e. radio, television, refrigerator, mobile telephone, cupboard, bicycle and motorcycle) was recorded as (Yes) “available and in working condition” or (No) “not available and/or not in working condition.” The TMD epidemiological questions were *Do you have pain in your temple, face, jaw or jaw joint once a week or more? Does it hurt once a week or more when you open your mouth or chew?* The response was either *yes or no* and positive answer to any of the two questions is considered affirmative to TMD diagnosis [50].

### Clinical examination

After an interview, clinical oral examinations were performed by the principal investigator (L.S). The child was examined under field conditions outside or inside the classroom sitting on a chair in natural day light, avoiding the direct sun light. When necessary, the teeth were cleaned and dried by sterile gauze and isolated by cotton rolls. Disposable mouth mirrors and sickle probes (No. 23 explorer or Shepherd’s hook) were used.

Oral hygiene was assessed using the Simplified Oral Hygiene Index (OHI-S) [51]. The scores were (0) for no plaque/calculus present, (1) for plaque or supra-gingival calculus covering not more than one third of the tooth surface, (2) for plaque or supra-gingival calculus covering more than one third but less than two thirds of the tooth surface, and (3) for plaque or supra-gingival calculus covering more than two thirds of the tooth surface. For each individual, the plaque and calculus scores were summed up and divided by total number of teeth examined to obtain the Simplified Debris Index (DI-S) and simplified calculus index (CI-S). The OHI-S was constructed by summing up the DI-S and CI-S. During analysis the OHI-S scores were dichotomized into 1 = good oral hygiene (OHI-S < 1) and 2 = poor oral hygiene (OHI-S ≥ 1).

Gingival health was assessed by Gingival Bleeding Index (GBI) [52]. Dental caries was assessed according to criteria specified by WHO, 2013 [53] and dental fluorosis was assessed by Thylstrup- Fejerskov - index (TF-index) [54]. Dental erosion on palatal and facial surfaces of maxillary anterior teeth was recorded according to Johansson et al. 1996 [55] and grading of first molar cuppings by Hasselkvist et al. [37]. Tooth wear was graded as a full mouth recording of occlusal/incisal surfaces according to Carlsson et al. [56].

### Statistical analysis

Data were analyzed using the Statistical Package for Social Sciences (SPSS) for PC, version 24 (IBM corporation, Armonk, NY, USA). STATA 14.2 (Stata corporation, Lakeway drive college station, Texas, USA) was used to adjust for the cluster effect of school. Principle component analysis was used to construct a socio-economic index categorized into wealth quintiles (1st quartile, 2nd quartile, 3rd quartile and 4th quartile implying the poorest, poorer, less poor and least poor respectively) and based on ownership of assets such as furniture and household characteristics including electricity, type of water source roof material and toilet types [48]. Descriptive statistics was carried out followed by bivariate analysis using cross tabulations and Pearson’s chi-square statistical test. Multiple variable logistic regression analysis (Odds Ratio and 95% CI) was carried out with all socio-demographic variables that were statistically significantly associated with oral hygiene status, gingival bleeding, dental caries, dental fluorosis, dental erosion/tooth wear, and TMD in Pearson’s chi-square test (unadjusted analysis) were included in the model simultaneously. For each outcome variables, there were two levels/categories used in the multiple variable logistic regression analysis. For instance, oral hygiene status was dichotomized into 0 = poor oral hygiene and 1 = good oral hygiene; gingival bleeding was dichotomized into 0 = without bleeding and 1 = with bleeding; dental caries was dichotomized into DMFT = 0 and DMFT > 0 and dental fluorosis was dichotomized into 0 = TF score 0–4 and 1 = TF score 5–9. In addition, Dental erosion was dichotomized into 0 = grade 0 and 1 = grade 1–4; tooth wear was dichotomized into 0 = grade 0 and 1 = grade 1–4; and TMD was dichotomized into 0 = without TMD symptoms and 1 = with TMD symptoms.

## Results

### Sample characteristics

A total of 989 grade 6 primary school adolescents were invited to participate in the study. Of those, 930 adolescents accepted to participate. Twenty-four (2.6%) children who attended the study were excluded during analysis due to too high or low age. Thus, the study included 906 children 12–17 years, mean age 13.4 years (SD 1.2) of which 56.1% were females. The final response rate was 91.6%. Among the participants, 52.9% were from Monduli district and the remaining from Longido district. Of the participants 79.6% were from the Maasai ethnic group and the remaining 20.4% from the non-Maasai group.

### Reliability

Duplicate clinical examinations (intra-examiner concordance, LS), 3 weeks apart, were carried out with 93 randomly selected participants. Analysis performed on duplicate examination records revealed Kappa value of 0.98, 0.87, and 0.69 for DMFT, TF-index, and dental erosion respectively. Higher Kappa values for DMFT and TF-index might have been contributed by the examiners common knowledge about the disease/condition in the population being rated [57].

### Socio-demographic characteristics by ethnicity

As depicted in Table 1, mothers who reported secondary school education level or above were 3.2% for the Maasais and 11.9% for the non-Maasais (*p* < 0.001). All socio-demographic characteristics except sex and age group differed statistically significantly between the two ethnic groups.

**Table 1:** Socio-demographic characteristics by ethnic groups

Variable	Categories	Maasai % (n)	Non-Maasai % (n)	*p*-value
District	Monduli	58.0 (418)	33.0 (61)	
	Longido	42.0 (303)	67.0 (124)	< 0.001*
Sex	Male	43.1 (311)	47.0 (87)	
	Female	56.9 (410)	53.0 (98)	0.341
Age group	12–14 years	86.5 (610)	90.3 (167)	
	15–17 years	13.5 (95)	9.7 (18)	0.173
Wealth index	1st quartile (poorest)	29.4 (209)	7.0 (13)	
	2nd quartile (very poor)	27.9 (199)	9.2 (17)	
	3rd quartile (less poor)	28.5 (203)	12.4 (23)	
	4th quartile (least poor	14.2 (101)	71.4 (132)	< 0.001*
Region of Birth	Arusha	99.0 (714)	78.4 (145)	
	Others	1.0 (7)	21.6 (40)	< 0.001*
Mother’s education	Low (≤ primary school)	96.8 (698)	88.1 (163)	
	High (≥ secondary school)	3.2 (23)	11.9 (22)	< 0.001*
Father’s education	Low (≤ primary school)	95.0 (685)	80.5 (149)	
	High (≥ secondary school)	5.0 (36)	19.5 (36)	< 0.001*
House ownership	Yes	98.6 (711)	80.0 (148)	
	No	1.4 (10)	20.0 (37)	< 0.001*
Number of children	1–5 children	45.1 (325)	63.8 (118)	
	6–14 children	54.9 (396)	36.2 (67)	< 0.001*

*Significant Pearson’s Chi-square test (*p* < 0.05)

Maasai *n* = 721, non-Maasai *n* = 185

### Prevalence, severity of oral diseases/conditions and correlated sociodemographic factors

The overall prevalence of poor oral hygiene (OHI-S ≥ 1) was 65.6% (good oral hygiene was 34.4%) and gingival bleeding was 40.9%. As depicted in Table 2 oral hygiene status and gingival bleeding varied statistically significantly according to district, sex, age group, birth region and ethnicity (*p* < 0.05). Good oral hygiene was more common among the non-Maasai adolescents (45.9%) than the Maasai adolescents (31.5%). A total of 27.4% of the males and 40.0% of the females had good oral hygiene (*p* < 0.05). Similarly, gingival bleeding was more common in Maasai adolescents (44.4%) than non-Maasai (27.6%) (*p* < 0.05).

**Table 2:** Frequency distribution of oral hygiene status and bleeding gums by socio-demographic factors

Variable	Oral hygiene status % Good (n with OHI-S < 1)	Gingival Bleeding % (n)
Prevalence (whole sample)	34.4 (312)	40.9 (371)
District		
Monduli	24.6 (118)	47.4 (227)
Longido	45.4 (194)*	33.7 (144)*
Sex		
Male	27.4 (109)	50.3 (200)
Female	40.0 (203)*	33.7 (171)*
Age group		
12–14	35.9 (279)	38.6 (300)
15–17	23.9 (27)*	56.6 (64)*
Region of Birth		
Arusha	33.6 (288)	42.3 (363)
Others	48.9 (23)*	17.0 (8)*
Ethnicity		
Maasai	31.5 (213)	44.4 (320)
Non-maasai	45.9 (85)*	27.6 (51)*
Wealth index		
1st quartile (poorest)	29.3 (65)	44.6 (99)
2nd quartile (very poor)	28.2 (61)	48.1 (104)
3rd quartile (less poor)	30.5 (69)	42.0(95)
4th quartile (least poor)	47.2 (110)*	30.9 (72)
Mother’s education		
Low (≤ primary school)	33.9 (292)	41.7 (359)
High (≥ secondary school)	44.4 (20)	26.7 (12)*
Father’s education		
Low (≤ primary school)	34.1 (285)	41.2 (344)
High (≥ secondary school)	38.9 (28)	37.5 (27)*

*Significant Pearson Chi-square test (*p* < 0.05)

Dental caries prevalence (DMFT> 0) was 8.8%. The overall mean DMFT was 0.13 (SD 0.5). The DMFT was composed of the decayed (D = 90.0%), missing teeth due to caries (M = 5.0%) and filled teeth (F = 0). Adolescents with both, decayed and missing teeth due to caries were 5.0%. The overall mean DMFS was 0.22 (SD 0.9). The mandibular first permanent molar was the most commonly registered tooth with dental caries (60.0%) and the occlusal surface was the most common registered site with caries (71.6%). As shown in Table 3, 7.4% of the Maasai adolescents had a DMFT> 0 and the corresponding figures for the non-Maasai was 14.6% (*p* < 0.05). Among the 12–14 year old adolescents, a total of 8.2 and 13.3% of the 15–17 year old adolescents had DMT > 0 (*p* < 0.05).

**Table 3:** Frequency distribution of dental caries experience (DMFT > 0) and severe dental fluorosis (TF grade 5–9) by socio-demographic factors

Variable	DMFT > 0 % (n)	Dental fluorosis % (n)
Prevalence (whole sample)	8.8 (80)	48.6 (440)
District		
Monduli	10.0 (48)	77.8 (339)
Longido	7.5 (32)	23.7 (101)*
Sex		
Male	9.5 (38)	47.2 (188)
Female	8.3 (42)	49.6 (252)
Age group		
12–14	8.2 (64)	46.3 (360)
15–17	13.3 (15)*	63.7 (72)*
Region of Birth		
Arusha	8.4 (72)	50.5 (434)
Others	17.0 (8)	12.8 (6)*
Ethnicity		
Maasai	7.4 (53)	52.1 (376)
Non-maasai	14.6 (27)*	34.6 (64)*
Wealth index		
1st quartile (poorest)	7.7 (17)	49.1 (109)
2nd quartile (very poor)	6.9 (15)	59.3 (128)
3rd quartile (less poor)	10.2 (23)	56.2 (127)
4th quartile (least poor)	10.7 (25)	30.9 (72)*
Mother’s education		
Low (≤ primary school)	8.8 (76)	49.2 (424)
High (≥ secondary school)	8.9 (4)	35.6 (16)
Father’s education		
Low (≤ primary school)	8.9 (74)	49.0 (409)
High (≥ secondary school)	8.3 (6)	43.1 (31)

*Significant Pearson Chi-square test (*p* < 0.05)

Dental fluorosis, TF grade 1–9, was recorded in 89.7% of the participants and more severe fluorosis TF-grade 5–9 in 48.6%. More severe dental fluorosis (TF grade 5–9) was more common among the Maasai than non-Masaai (52.1 and 34.6% respectively, *p* < 0.05). About 51% of the adolescents born in Arusha region was registered with severe dental fluorosis TF score 5–9, while only 12.8% of the adolescents born outside Arusha region (*p* < 0.05) had so.

**Fig. 1:**
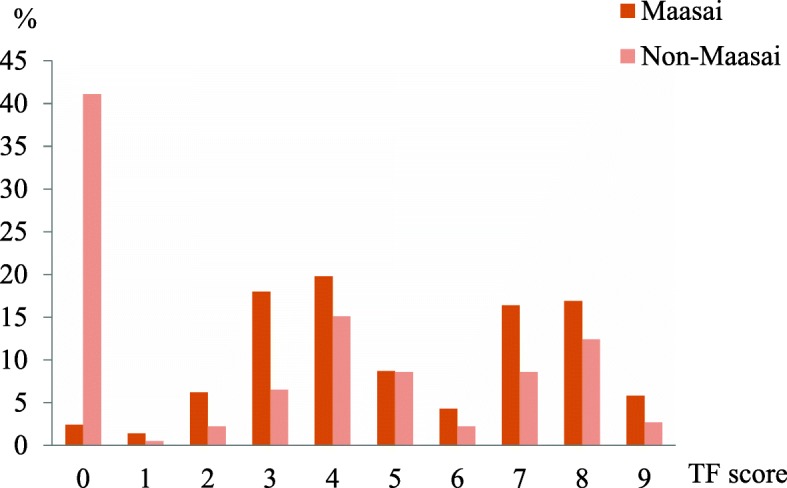
Percentage distributions of children with dental fluorosis according to maximum TF score per subject by ethnic groups. Maasai *n* = 721; non-Maasai *n* = 185

Figure 1 shows the percentage distributions of adolescents according to maximum TF score per subject by ethnic groups. Dental fluorosis, TF grade 1–9, was more common and more severe among the Maasai adolescents (97.6%) than the non-Maasai (58.9%).

Dental erosion extending into dentine (grade 3–4) was registered in 1.9% (grade 1–4 was 30.0%) of the participants and tooth wear extending into dentine (grade 2–4) in 16.5% (grade 1–4 was 44.3%). As shown in Table 4 dental erosion was less common among the Maasai- than the non-Maasai- adolescents since 26.8 and 43.8%, respectively showed any grade of erosion (grade 1–4) (*p* < 0.05). Furthermore, dental erosion was registered among 24.0% of the adolescents from Monduli districts and 36.8% of the adolescents from Longido districts (*p* < 0.05). However, none of them was registered with the most severe grade of dental erosion (grade 4). In general, the pattern of tooth wear was more similar across the ethnic groups and 44.9% of the Maasais and 50.3% of the non-Maasais was registered with some type of tooth wear (*p* > 0.05).

**Table 4:** Frequency distribution of dental erosion (grade 1–4), tooth wear (grade 1–4) and TMD by socio-demographic factors

Variable	Dental erosion % (n)	Tooth wear % (n)	TMD > 0 % (n)
Prevalence (whole sample)	30.0 (372)	44.3 (401)	11.8 (107)
District			
Monduli	24.0 (115)	42.8 (205)	18.0 (86)
Longido	36.8 (157)*	45.9 (196)	4.9 (21)*
Sex			
Male	28.1 (112)	46.0 (183)	12.3 (49)
Female	31.5 (160)	42.9 (218)	11.4 (58)
Age group			
12–14	30.4 (236)	43.8 (340)	11.2 (87)
15–17	28.3 (32)	48.7 (55)	17.7 (20)*
Region of Birth			
Arusha	29.2 (251)	44.7 (384)	11.9 (102)
Others	47.7 (21)*	36.2 (17)	10.6 (5)
Ethnicity			
Maasai	26.8 (193)	44.9 (324)	11.2 (81)
Non-maasai	43.8 (81)*	50.3 (92)	14.1 (26)
Wealth index			
1st quartile (poorest)	29.7 (66)	28.6 (113)	12.2 (27)
2nd quartile (very poor)	25.5 (55)	23.0 (91)	8.8 (19)
3rd quartile (less poor)	20.8 (47)	21.5 (85)	13.3 (30)
4th quartile (least poor)	42.9 (100)*	26.8 (106)*	12.9 (30)
Mother’s education			
Low (≤ primary school)	28.8 (248)	44.5 (383)	10.8 (93)
High (≥ secondary school)	53.3 (24)*	40.0 (18)	31.1 (14)*
Father’s education			
Low (≤ primary school)	29.4 (245)	44.2 (369)	11.6 (97)
High (≥ secondary school)	37.5 (27)	44.4 (32)	13.9 (10)

*Significant Pearson Chi-square test (*p* < 0.05)

TMD pain (TMD-p) was found in 11.8% of the participants. The prevalence of TMD among Maasais and non-Maasais was 11.2 and 14.1% respectively (*p* > 0.05).

Socio-demographic variables found statistically significantly associated with oral diseases and problems in unadjusted analysis (Tables 2, 3 and 4) were entered simultaneously, into seven separate multiple variable logistic regression models in order to investigate the likelihood of having good oral hygiene, gingival bleeding, dental caries experience (DMFT> 0), dental fluorosis TF grade 5–9, dental erosion (grade 1–4), tooth wear (grade 1–4), and TMD (2Q/TMD > 0). As shown in Table 5, adolescents from Longido district were 2.6 times (OR = 2.6, CI 1.6–4.4) more likely to have good oral hygiene compared to adolescents from Monduli district. Females were 2.0 (OR = 2.0, CI 1.4–2.5) times more likely to have good oral hygiene compared to males. Adolescents from Longido district, females and non Maasai were respectively 0.6-times (OR = 0.6, CI 0.4–0.8), 0.5-times (OR = 0.5, CI 0.4–0.6) and 0.6 (0.4–0.9) times less likely to have gingival bleeding compared to their counterparts in Monduli district, males and Maasais. Adolescents born within the Arusha region and older age groups were 2.0-times (OR = 2.0, CI 1.2–3.3) and 1.6 (1.0–2.5) more likely to have gingival bleeding compared to those born outside Arusha and younger age group. Statistically significant two-way interactions occurred between ethnicity and wealth index on oral hygiene status whereby adolescents from non Maasais and least poor families were 2.8 (OR = 2.8, CI 1.1–7.2) times more likely to have good oral hygiene compared to those from Maasais and most poor families. Female adolescents from non Maasai were 2.5 (OR = 2.5, CI 1.2–5.4) times more likely to have gingival bleeding compared to males from Maasais group. As shown in Table 6, older aged (OR = 2.0, CI 1.1–3.5) and non-Maasai adolescents (OR = 2.2, CI 1.1–4.1) were more likely to have DMFT> 0 compared to the younger aged and Maasai adolescents, respectively. Adolescents from Longido district (OR = 0.1, CI 0.1–0.3) were less likely to have dental fluorosis TF grade 5–9 than children from Monduli district. Moreover, adolescents born within the Arusha region (OR = 3.2, CI 1.0–10.2) were 3.2-times more likely to have dental fluorosis TF grade 5–9 than those born outside the Arusha region . Statistically significant two way interaction occurred between ethnicity and district on DMFT, and between ethnicity and wealth index on dental fluorosis. Adolescents from non Maasai and Longido district were 3.9 (OR = 3.9, CI 1.3–12.2) times more likely to have DMFT> 0 in comparison with those from Maasai and Monduli district. Also non Maasai adolescents and being from least poor families were 0.1 (OR = 0.1, CI 0.04–0.3) times less likely to have dental fluorosis compared to Maasais and from most poor families. As shown in Table 7, non-Maasai adolescents and adolescents of mothers with high education level were 2.0 (OR = 2.0, CI 1.3–3.2) and 2.3 (OR = 2.3, CI 1.3–4.0) times more likely to have dental erosion than Maasai adolescents and adolescents from mothers with low education level, respectively. No cuppings were observed on occlusal surfaces of first molars. Considering TMD, adolescents from Longido district (OR = 0.2, CI 0.1–0.4) were 0.2-times less likely to have TMD compared to those from Monduli district. Also adolescents from mothers with high education (OR = 5.1, CI 2.5–10.2) were more likely to have TMD compared to those from mothers with low education. Regarding TMD, statistically significant two way interaction occurred between ethnicity and district where non Maasai adolescents and being from Longido district were 3.4 (OR = 3.4, CI 1.1–10.4) times more likely to have TMD compared to those from Maasai and being from Monduli district.

**Table 5:** Oral hygiene status and gingival bleeding regressed on socio-demographic characteristics and statistically significant interactions

Variable	Oral hygiene status (Good oral hygiene)		Gingival bleeding	
	OR (95% CI)	*P*	OR (95% CI)	*P*
District				
Monduli	1		1	
Longido	2.6 (1.6–4.4)	< 0.001	0.6 (0.4–0.8)	0.002
Sex				
Male	1		1	
Female	2.0 (1.4–2.5)	< 0.001	0.5 (0.4–0.6)	< 0.001
Age group				
12–14 years	1		1	
15–17 years	0.8 (0.4–1.5)	0.519	1.6 (1.0–2.5)	0.053
Region of birth				
Others	1		1	
Arusha	1.0 (0.7–1.5)	0.794	2.0 (1.2–3.3)	0.005
Ethnic groups				
Maasai	1		1	
Non-Maasai	1.4 (0.9–2.4)	0.140	0.6 (0.4–0.9)	0.008
Wealth index				
Most poor	1			
Least poor	1.3 (0.8–2.0)	0.323		
Ethnicity by wealth Index				
Maasai x most poor	1			
Non Maasai x least poor	2.8 (1.1–7.2)	0.038		
Ethnicity by sex				
Maasai x Males			1	
Non Maasai x Female			2.5 (1.2–5.4)	0.019

Adjusted odds ratios (OR) and 95% confidence intervals (CI)

**Table 6:** Dental caries and dental fluorosis regressed on socio-demographic characteristics and statistically significant interactions

Variable	Dental caries (DMFT > 0)		Dental fluorosis (TF grade 5–9)	
	OR (95% CI)	*P*	OR (95% CI)	*P*
District				
Monduli			1	
Longido			0.1 (0.1–0.3)	< 0.001
Sex				
Male				
Female				
Age group				
12–14 years	1		1	
15–17 years	2.0 (1.1–3.5)	0.017	1.4 (0.9–2.3)	0.171
Region of birth				
Others			1	
Arusha			3.2 (1.0–10.2)	0.044
Ethnic groups				
Maasai	1		1	
Non-Maasai	2.2 (1.1–4.1)	0.018	0.9 (0.5–1.8)	0.767
Wealth index				
Most poor			1	
Least poor			1.0 (0.7–1.4)	0.880
Ethnicity by district				
Maasai x Monduli	1			
Non-Maasai x Longido	3.9 (1.3–12.2)	0.018		
Ethnicity by wealth index				
Maasai x most poor			1	
Non-Maasai x Least poor			0.1 (0.04–0.3)	< 0.001

Adjusted odds ratios (OR) and 95% confidence intervals (CI)

**Table 7:** Dental erosion, tooth wear and TMD regressed on socio-demographic characteristics and statistically significant interactions

Variable	Dental erosion (Grade 1–3)		Tooth wear (Grade 1–4)		TMD (2Q/TMD > 0)	
	OR (95% CI)	*P*	OR (95% CI)	*P*	OR (95% CI)	*P*
District						
Monduli	1				1	
Longido	1.6 (0.9–3.2)	0.140			0.2 (0.1–0.4)	< 0.001
Sex						
Female					1	
Male					1.2 (0.7–2.2)	0.483
Age group						
12–14 years					1	
15–17 years					1.3 (0.8–2.2)	0.251
Region of birth						
Others	1		1			
Arusha	1.1 (0.5–2.3)	0.897	1.3 (0.9–1.9)	0.129		
Ethnic groups						
Maasai	1				1	
Non maasai	2.0 (1.3–3.2)	0.003			1.6 (0.9–2.9)	0.096
Wealth index						
Most poor	1		1			
Least poor	0.9 (0.6–1.2)	0.455	0.8 (0.7–1.1)	0.157		
Mother’s education						
Low (≤ primary school)	1				1	
High (≥ secondary school)	2.3 (1.3–4.0)	0.002			5.1 (2.5–10.2)	< 0.001
Ethnicity by district						
Maasai x Monduli					1	
Non Maasai x Longido					3.4 (1.1–10.4)	0.034

Adjusted odds ratios (OR) and 95% confidence intervals (CI)

## Discussion

To our knowledge, this is the first study reporting on the prevalence, severity and socio-demographic distribution of oral diseases and conditions affecting Tanzanian school adolescents living in Maasai population areas of Monduli and Longido districts. According to the present findings, poor oral hygiene and gingival bleeding on gentle probing was common in the study group as a whole, whereas both severity and prevalence of dental caries prevalence seemed to be quite marginal. Moreover, about half the study group presented with sever dental fluorosis whereas the presence of erosion, tooth wear and TMD symptoms were rare.

In this study, poor oral hygiene was found to be more common than in other comparable studies from sub-Saharan Africa since about 66% of the school adolescents investigated showed bad oral hygiene compared to only 32–45% reported earlier for instance in Tanzania and Nigeria [58–60]. Consistent with the sex distribution of oral hygiene observed in this study, where girls were twice as likely as boys to have good oral hygiene status, this finding is in agreement with several other studies from Nigeria (10–19 year olds) [61], Kenya (12 year olds) [62] and India (7–12 year olds) [63]. Contrary to these findings, one urban study in Tanzania (15 year olds) found that poor oral hygiene status was equally distributed between girls and boys [60]. A large proportion of the adolescents (41%) investigated showed gingival bleeding on gentle probing, a sign of gingivitis. This prevalence is lower than the one reported in 12 year old children in Uganda where about 54% of the participants presented with gingival bleeding [17].

The mean DMFT (0.13) in our study was low and in agreement with other studies from East Africa [19–21]. As expected, the prevalence of dental caries in this study was significantly higher in older than in younger age groups [15–17, 64, 65]. The decayed component (D) of the DMFT- index in our study constituted 90%, and the missing component (M) was 10%. It is noteworthy that none of the participants in this study was diagnosed with any type of dental restoration. The lack of dental restorations has earlier been reported on from other studies in sub-Saharan Africa [2, 20, 66] and may be an indicator of limited professional oral health services and poor socioeconomic situation prevailing in this area. The finding that Maasai adolescents had less dental caries compared to non-Maasai adolescents, is also in agreement with previous findings dateing as far back as 1931 but also 1997 [22, 23].

As expected, due to the high fluoride content in the drinking water [67–69], severe dental fluorosis (TF grade 5–9) was common and presented in nearly half of the participants (48.6%). This is also in accordance with earlier findings from Kenya [32] but contrary to other studies from Tanzania and Ethiopia [33, 70] where a lower prevalence of severe dental fluorosis (10–34%) has been reported. Identified sociodemographic covariates of dental fluorosis were location and region of birth. In this regard, previous studies from the Arusha region reported a fluoride concentration between 0.1–78.09 mg/l in drinking water [67, 68].

In the present study, dental erosion extending in to dentine was found only in 1.9% of the adolescents. This is lower than that found in other comparable studies from Saudi Arabia and Sweden which reported prevalence of 11.9 to 26.0% [36, 37]. The rural environment in which the adolescents in this study from both districts live in combination with a restricted economic situation and a reduced availability to shops might be limiting the access to erosive conducive products/challenges. Non-Maasai adolescents were more prone to have dental erosion than Maasai adolescents. This might be attributed to differences in exposure to risk factors for erosion between the two ethnic groups. Adolescents whose mothers had higher levels of education had an elevated risk to develop dental erosion than children whose mothers had low education. This finding is in agreement with some studies [71, 72] but in contradiction to other studies [73, 74]. The relationship between the mother’s high level of education and the presence of dental erosion in the children in this study may be due to the wealth and lifestyle of such a family and thereby greater chances for the children to consume erosive conducive products.

This study revealed that 16.5% of the adolescents had tooth wear extending into dentine. This is contrary to findings from England where a higher prevalence (30.0–53.0%) has been reported [75, 76] and also contrary to findings from China, Nigeria, which found lower prevalence rates (1.9–8.5%) [77, 78]. Regression analysis revealed no sociodemographic variable that was significantly correlated with tooth wear. Thus, non of the socio-demographic factors of importance for the development of tooth wear was included in the present analysis.

Comparing studies using a similar diagnostic system (TMD-p, RDC/TMD), the prevalence of adolescents with TMD pain in this study was 11.8% which is slightly higher than in other previous studies in Sweden and United States of America [45, 79–81], but also lower than other previous studies in Saudi Arabia and China [46, 79]. Psychosocial factors, for example stress related behaviors, are considered to be a common cause for development of TMD [82]. It was expected that in a nomadic society, this impact would be low and thus lessen the occurrence of reported TMD, but instead the prevalence of TMD was higher than in comparable studies performed in Western societies. This variation in TMD magnitude could be because of the differences related to heterogeneous age groups, the sample size and the setting of sample selection [83]. This study identified adolescents from Monduli district and adolescents of mothers with high education to be more likely to have TMD pain. This may imply that environmental and cultural factors may have a great role in the development of TMD among adolescents. One American study reported similar findings, that the geographical study site was independently associated with TMD [84]. However, our finding is contrary to the findings from China, which found that children and adolescents with lower parental educational levels or household income, had higher rates of TMD pain [79]. Similar findings on correlation between low educational level and other types of pain has also been reported on [85, 86]. The correlation between high education level of the mother and TMD in our study may be due to subgroup differences in emotional responsivity to chronic pain as well as pain intensity within a group of individuals [86]. However, further investigations are needed to clarify the association between mother’s education level and TMD in this particular society.

### Study limitations

The cross-sectional method utilized in data collection, has some drawbacks as it is difficult to establish a causal relationship. The information on oral health was collected by interviews/clinical examination conducted by trained medical/dental personnel. Information bias, recall bias and social desirability bias might have been introduced due to the self-report method employed. In addition, fewer male than female adolescents and school non-attenders in our total study sample, might have contributed to a selection bias that could have affected the study findings and generalization. Relatively fewer males than females in our study sample might be due to the fact that, in their setting male adolescents are responsible for taking care of their livestock, as a result some do not attend schools at all and some start attending schools very late age-wise. Although the methods used in this study have been utilized in other studies in East Africa [60, 87], precautious interpretation should be employed in extrapolating these findings into other societies in Tanzania, but it can be useful in other Maasai population areas in the country, especially for adolescents attending primary schools. There is a need for conducting longitudinal studies in order to further assess the sociodemographic risk indicators in this society and to explore the direction of the relationships identified.

## Conclusion

The study showed that oral diseases/conditions like dental caries and dental erosion were less common, but gingival bleeding, dental fluorosis, tooth wear and TMD were common findings in adolescents attending primary schools in the Maasai population areas in Tanzania. Notable differences between Maasai and non-Maasai ethnic groups and certain correlations to sociodemographic factors were detected. Our findings can be utilized by policy makers in the planning of oral health programs in all public primary schools of Maasai population areas of Tanzania in order to address oral diseases/conditions in this area.

## Abbreviations

2Q/TMD: 2 epidemiological Questions on Temporomandibular Disorder
CI: Confidence Interval
CI-S: Simplified Calculus Index
DI-S: Simplified Debris Index
DMFS: Decayed Missing Filled Surfaces
DMFT: Decayed Missing Filled Tooth
DMT: Decayed Missing Tooth
GBI: Gingival Bleeding Index
OHI-S: Simplified Oral Hygiene Index
OR: Odds Ratio
RDC: Research Diagnostic Criteria
SD: Standard Deviation
SPSS: Statistical Package for Social Sciences
TF-index: Thylstrup-Fejerskov-index
TMD: Temporomandibular Disorder
TMDp: Temporomandibular Disorder pain
WHO: World Health Organisation
